# The Novel Protein ADAMTS16 Promotes Gastric Carcinogenesis by Targeting IFI27 through the NF-κb Signaling Pathway

**DOI:** 10.3390/ijms231911022

**Published:** 2022-09-20

**Authors:** Tuoyang Li, Junyi Zhou, Yingming Jiang, Yandong Zhao, Jintuan Huang, Weiyao Li, Zhenze Huang, Zijian Chen, Xiaocheng Tang, Hao Chen, Zuli Yang

**Affiliations:** 1 Department of Gastrointestinal Surgery, the Sixth Affiliated Hospital, Sun Yat-sen University, 26 Yuancun Erheng Rd, Guangzhou 510655, China; 2 Guangdong Provincial Key Laboratory of Colorectal and Pelvic Floor Diseases, the Sixth Affiliated Hospital, Sun Yat-sen University, 26 Yuancun Erheng Rd, Guangzhou 510655, China; 3 Department of Pathology, the Sixth Affiliated Hospital, Sun Yat-sen University, Guangzhou 510655, China

**Keywords:** ADAMTS16, gastric cancer, tumor promoter, IFI27, NF-κB

## Abstract

A disintegrin and metalloproteinase with thrombospondin motifs 16 (ADAMTS16) has been reported to be involved in the pathogenesis of solid cancers. However, its role in gastric cancer (GC) is unclear. In this study, the role of ADAMTS16 in gastric cancer was investigated. The effects of ADAMTS16 on cell migration, invasion, and proliferation were investigated by functional experiments in vivo and in vitro. Downstream signal pathways of ADAMTS16 were confirmed by using bioinformatics analysis, co-immunoprecipitation, and immunofluorescence. Meanwhile, bioinformatics analysis, qRT-PCR, western blot, and dual-luciferase reporter gene analysis assays were used to identify ADAMTS16 targets. The expression of ADAMTS16 in GC was analyzed in public datasets. The expression of ADAMTS16 and its correlations with the clinical characteristics of GC were investigated by immunohistochemistry. Ectopic ADAMTS16 expression significantly promoted tumor cell migration, invasion, and growth. Bioinformatics analysis and western blot showed that ADAMTS16 upregulated the IFI27 protein through the NF-κb pathway, which was confirmed by immunofluorescence and western blot. Dual-luciferase reporter gene analysis identified a binding site between P65 and IFI27 that may be directly involved in the transcriptional regulation of IFI27. IFI27 knockdown reversed the promoting effect of ADAMTS16 on cell invasion, migration, and proliferation indicating that ADAMTS16 acts on GC cells by targeting the NF-κb/IFI27 axis. ADAMTS16 was associated with poor prognosis in clinical characteristics. ADAMTS16 promotes cell migration, invasion, and proliferation by targeting IFI27 through the NF-κB pathway and is a potential progressive and survival biomarker of GC.

## 1. Introduction

Gastric cancer (GC) is one of the most common cancers in the world, with more than 1 million new cases and an estimated 769,000 deaths in 2020, and ranks fifth and fourth in incidence and mortality, respectively, among malignant tumors worldwide [1]. As of 2015, the incidence and mortality rate of gastric cancer in China had risen to second only to lung cancer [2]. In recent years, despite significant progress in surgery, chemotherapy, targeted therapy, and biological therapy, the prognosis of cancer patients has remained poor [3,4]. Therefore, identifying novel factors and better insight into the mechanisms underlying gastric cancer would assist with the development of more effective diagnostic and/or therapeutic strategies.

ADAMTS16 is a member of the ADAMTS (a disintegrin and metalloproteinase with thrombospondin motifs) protein family, which includes a propeptide region, a metalloproteinase domain, a disintegrin-like domain, and a thrombospondin type 1 (TS) motif [5]. Respective members of this family are distinguished by the different numbers of C-terminal TS motifs, and some have unique C-terminal domains. It was expressed in human organs ubiquitously with a particularly abundant distribution in human fetus lung, ovary, kidney, and adult brain [6]. ADAMTS16 has been proven to be associated with human diseases. Pyun JA et al. discovered that ADAMTS16 plays a critical role in premature ovarian failure by interacting with thyroglobulin [7]; Yao Y et al. revealed that ADAMTS16 promotes fibrosis and dysfunction of the pressure-overloaded heart by acting on TGF-beta [8]. In recent studies, ADAMTS16 may be involved in tumor biological procession [9,10,11,12]. Cakmak et al. reported that ADAMTS16 was downregulated in a chondrosarcoma cell [11]. In colorectal cancer (CRC), ADAMTS16 was hypermethylated and high expression of ADAMTS16 restrained cancer cell proliferation [9,13]. Meanwhile, the mutation of ADAMTS16 altered the sensibility of ovarian cancer to platinum-based chemotherapy [10]. On the contrary, knockdown ADAMTS16 expression in esophageal squamous cell carcinoma could inhibit cell invasion and proliferation [12]. However, rare evidence exists regarding ADAMTS16’s precise functionality and underlying molecular mechanism in the pathology of GC.

In this study, we first aimed to comprehensively investigate the effects of ADAMTS16 and its associated mechanisms in GC. We next aimed to correlate ADAMTS16 with GC patient prognosis and clinicopathological features to determine whether it can be used as a prognostic predictor. Based on these findings, our goal is to develop a novel biomarker for future GC diagnosis and treatment.

## 2. Results

### 2.1. Ectopic ADAMTS16 Expression Promotes GC Cell Migration and Invasion In Vitro

**Figure 1:**
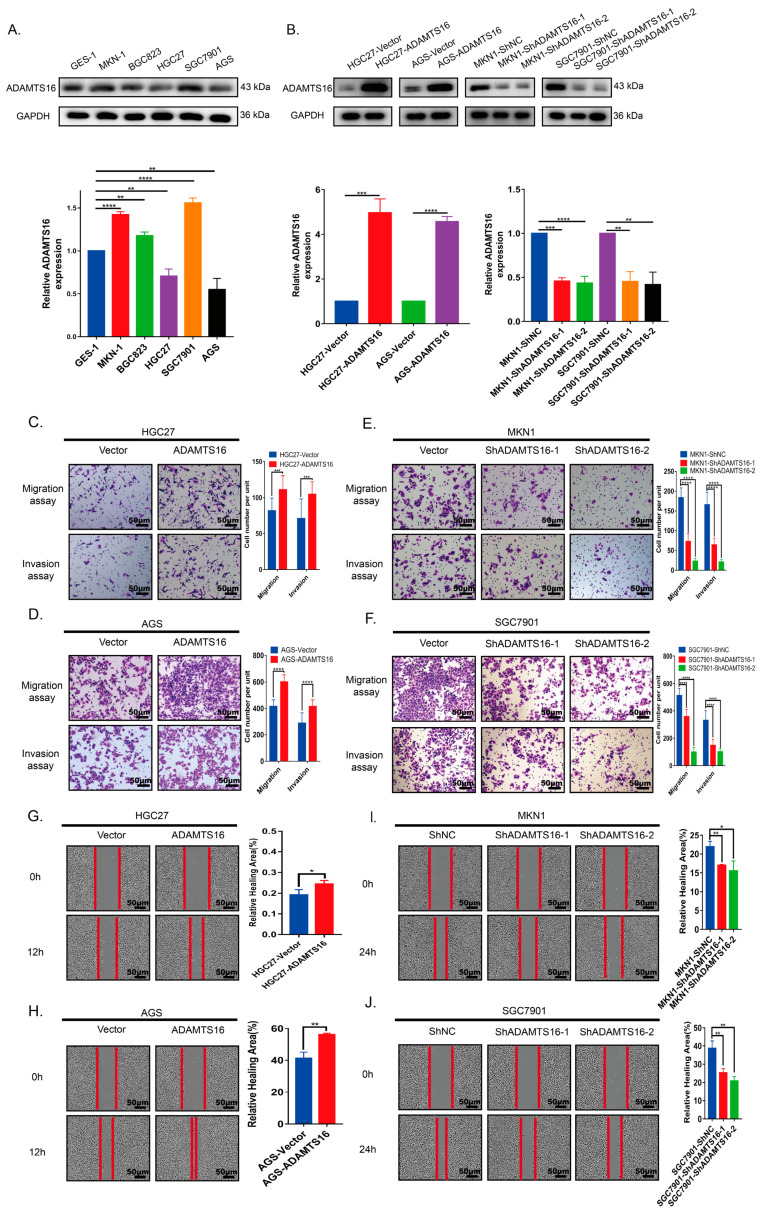
ADAMTS16 promotes GC cell migration and invasion in vitro. (**A**) Quantitation and western blot analysis of ADAMTS16 protein expression in five GC cell lines (MKN1, BGC823, HGC27, SGC7901, and AGS) and normal gastric epithelial cells (GES1). (**B**) The expression of ADAMTS16 in stably transfected cells was confirmed by quantitation and western blot analysis. (**C**–**F**) Transwell assays were used to assess cell migration and invasion in the indicated cell lines (original magnification: 200×). Scale bar, 50 µm. (**G**–**J**) Representative images of wound healing assays at indicated times (original magnification: 200×). The data are shown as means ± standard deviations. * *p* < 0.05, ** *p* < 0.01, *** *p* < 0.001, **** *p* < 0.0001. ns, not significant.

We used western blotting to detect the endogenous expression of ADAMTS16 in GC cell lines (Figure 1A). HGC27 and AGS cell lines with low endogenous ADAMTS16 expression were selected to construct lentiviral vector-transfected cells stably overexpressing ADAMTS16 (Figure 1A), while MKN1 and SGC7901 cell lines with high endogenous ADAMTS16 expression were selected to construct lentiviral vector-transfected cells with stable knockdown of ADAMTS16 (Figure 1A). Stable cell construction is shown in Figure 1B. Transwell assays indicated that ADAMTS16 overexpression promoted HGC27 (*p* < 0.001, Figure 1C) and AGS (*p* < 0.0001, Figure 1D) cells migration more than the control group. Similarly, invasion assays showed that ectopic expression of ADAMTS16 markedly enhanced cell invasive ability in both HGC27 (*p* < 0.001, Figure 1C) and AGS cells (*p* < 0.0001, Figure 1D). Conversely, as predicted, the inhibition of ADAMTS16 expression decreased MKN1 and SGC7901 cells migration and invasion (both *p* < 0.0001, Figure 1E,F). Moreover, wound healing assays demonstrated that ADAMTS16 overexpression promoted cell horizontal migration in GC cells HGC27 (*p* < 0.05, Figure 1G) and AGS (*p* < 0.01, Figure 1H), whereas ADAMTS16 downregulation inhibited it in MKN1 (ShNC vs. ShADAMTS16-1, *p* < 0.01; ShNC vs. ShADAMTS16-2, *p* < 0.05; Figure 1I) and SGC7901 (both *p* < 0.01, Figure 1J).

### 2.2. Ectopic ADAMTS16 Expression Stimulates GC Cell Growth In Vitro and In Vivo

**Figure 2:**
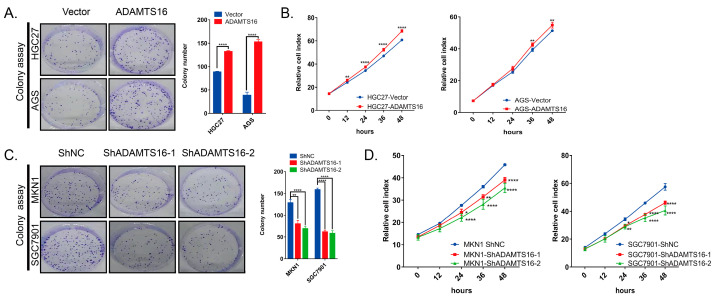

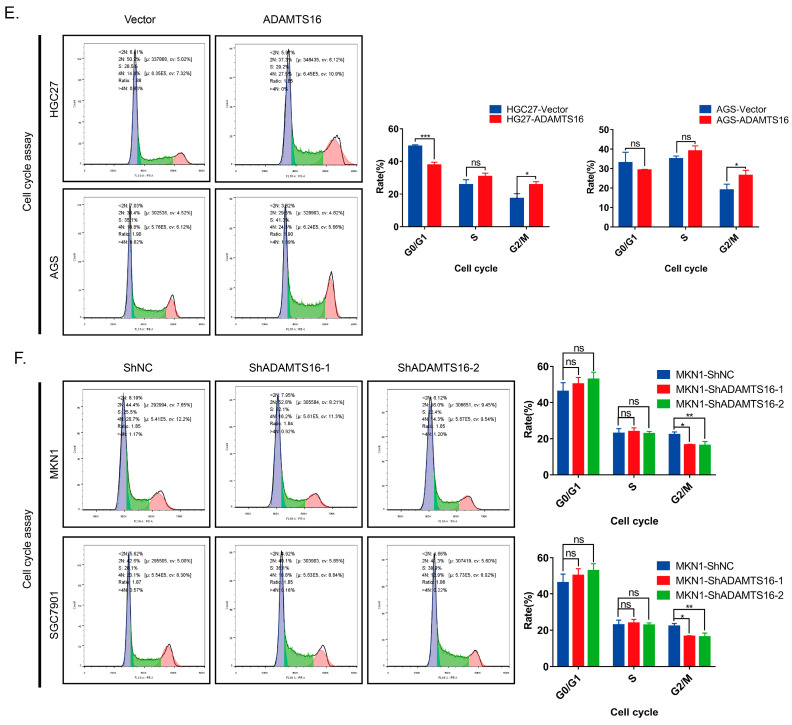

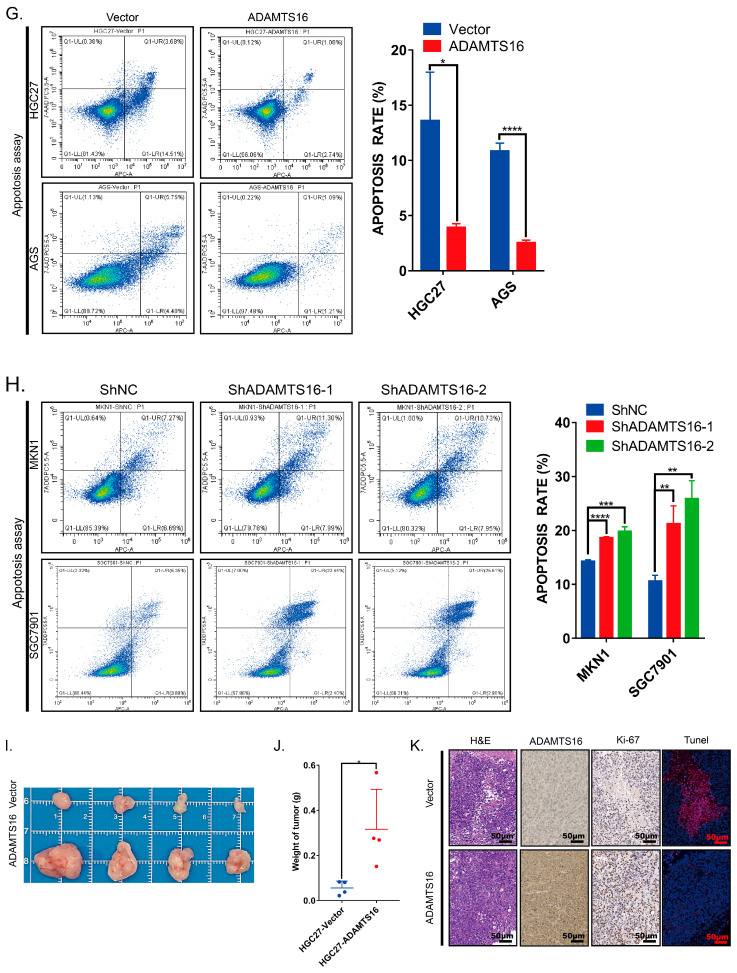
Effects of ADAMTS16 expression on GC cell and growth in vitro and in vivo. (**A**–**D**) Representative colony formation assay images and quantitative analysis of cell proliferation. (**E**,**F**) Flow cytometry showed that ADAMTS16 expression in GC cell lines induced G2/M phase arrest. (**G**,**H**) Flow cytometry was used to reveal the effects of ADAMTS16 expression on GC cell apoptosis arrest. (**I**–**K**) Effect of ADAMTS16 expression on tumor growth in nude mice and representative immunohistochemistry images of ADAMTS16, Ki67 and TUNEL in tumor tissues. Scale bar, 50 µm. The data are shown as means ± standard deviations. * *p* < 0.05, ** *p* < 0.01, *** *p* < 0.001, **** *p* < 0.0001. ns, not significant.

ADAMTS16 overexpression significantly enhanced HGC27 and AGS clonogenicity compared to the controls by clone formation assays (both *p* < 0.0001, Figure 2A). As expected, the cell proliferation significantly increased for HGC27 at 12 h (HGC27-Vector vs. HGC27-ADAMTS16, *p* < 0.01; Figure 2B) and AGS at 36 h (AGS-Vector vs. AGS-ADAMTS16, *p* < 0.01; Figure 2B). Conversely, ADAMTS16 knockdown reduced the clonogenic ability of MKN1 (ShNC vs. ShADAMTS16-1, *p* < 0.01; ShNC vs. ShADAMTS16-2, *p* < 0.0001; Figure 2C) and SGC7901 (ShNC vs. ShADAMTS16-1, *p* < 0.0001; ShNC vs. ShADAMTS16-2, *p* < 0.0001; Figure 2C). Meanwhile, MKN1 (ShNC vs. ShADAMTS16-1, *p* < 0.05; ShNC vs. ShADAMTS16-2, *p* < 0.0001; Figure 2D) and SGC7901 (ShNC vs. ShADAMTS16-1, *p* < 0.05; ShNC vs. ShADAMTS16-2, *p* < 0.01; Figure 2D) cell proliferation were restrained at 24 h, respectively. To further explore the mechanism by which ADAMTS16 promotes cell growth, we analyzed cell cycle and apoptosis by flow cytometry. HGC27 and AGS overexpressing ADAMTS16 had more cells distributed in the G2/M phase than the control group (HGC27-Vector vs. HGC27-ADAMTS16, *p* < 0.05; AGS-Vector vs. AGS-ADAMTS16, *p* < 0.05; Figure 2E). In contrast, in ADAMTS16 knockdown MKN1 (ShNC vs. ShADAMTS16-1, *p* < 0.05; ShNC vs. ShADAMTS16-2, *p* < 0.01; Figure 2F) and SGC7901 (ShNC vs. ShADAMTS16-1, *p* < 0.01; ShNC vs. ShADAMTS16-2, *p* < 0.01; Figure 2F) cell lines, the proportion of cells in the G2/M phase decreased compared with the control group. As expected, in apoptosis assays, ectopic expression of ADAMTS16 significantly decreased the proportion of apoptosis in HGC27 and AGS cells (HGC27-Vector vs. HGC27-ADAMTS16, *p* < 0.05; AGS-Vector vs. AGS-ADAMTS16, *p* < 0.0001; Figure 2G), while silencing expression of ADAMTS16 dramatically increased the proportion of apoptosis in MKN1 (ShNC vs. ShADAMTS16-1, *p* < 0.0001; ShNC vs. ShADAMTS16-2, *p* < 0.001; Figure 2H) and SGC7901 (ShNC vs. ShADAMTS16-1, *p* < 0.01; ShNC vs. ShADAMTS16-2, *p* < 0.01; Figure 2H) cells. These results revealed that ADAMTS16 upregulation stimulates cell growth by promoting cell proliferation and inhibiting cell apoptosis.

The in vivo experiment results were consistent with the in vitro results. ADAMTS16 tumors grew faster and larger than vector tumors (Figure 2I,J). Ki67 and TUNEL showed that ADAMTS16 promoted tumor cell proliferation (Figure 2K).

### 2.3. ADAMTS16 Promotes Cell Migration, Invasion and Proliferation Via the NF-κB/IFI27 Axis

**Figure 3:**
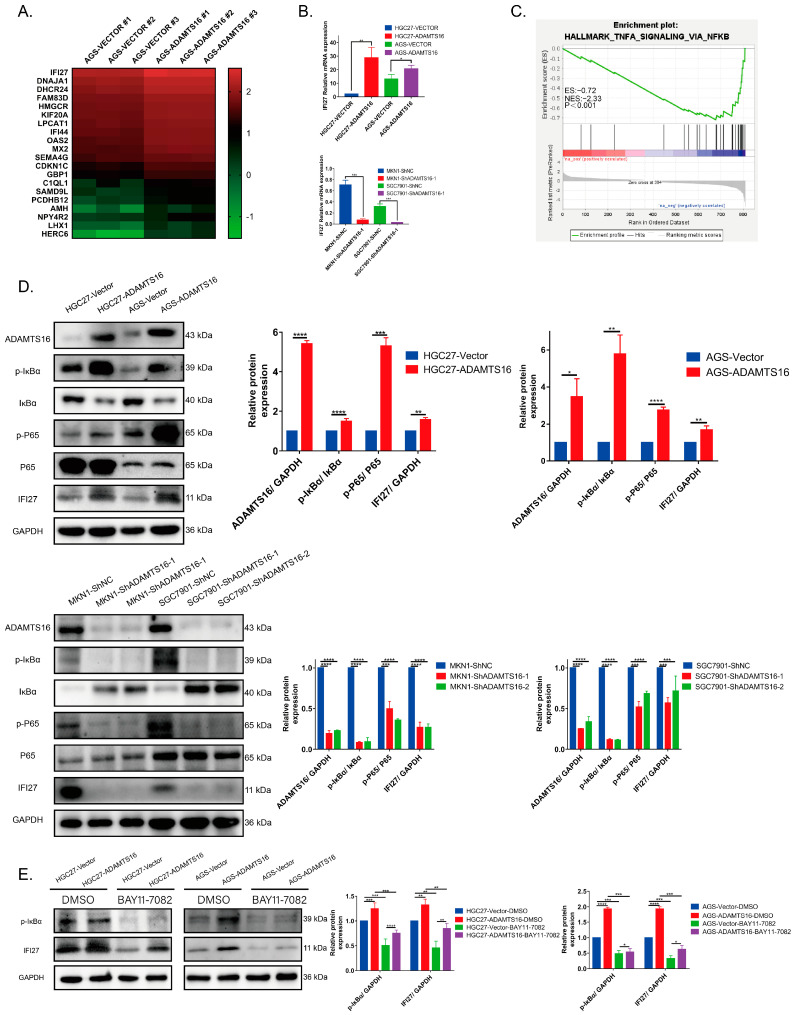

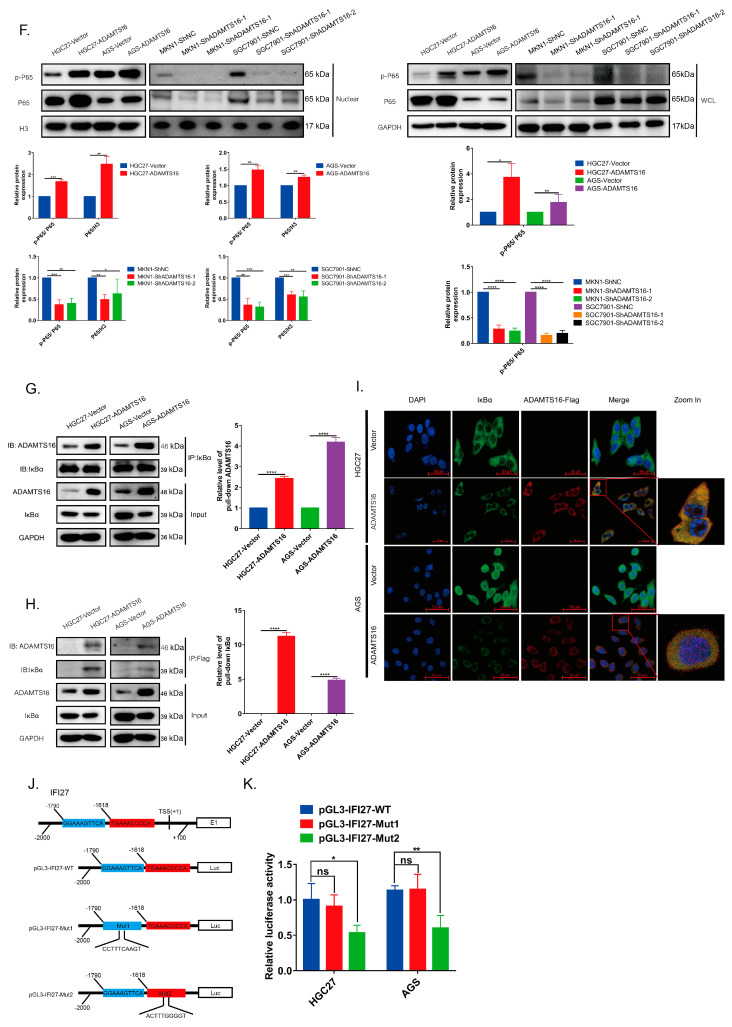
Ectopic ADAMTS16 expression promotes GC cell invasion and migration via the NF-κΒ/IFI27 axis. (**A**) Heat map analysis showed altered genes in AGS-vector and AGS-ADAMTS16 cells. (**B**) Quantitative real-time polymerase chain reaction was performed to detect IFI27 mRNA expression in stably ADAMTS16-expressing cell lines. (**C**) Gene set enrichment analysis showed enrichment of ADAMTS16-associated genes in the HALLMARK_TNF_SIGNALING–VIA_NFKB pathway. (**D**) Western blot and quantitation of phospho-IκBα, IκBα, phospho-P65, P65, and IFI27 in stably transfected GC cell lines. (**E**) Western blot and quantitation were performed to analyze the expression of IFI27 in stably transfected HGC27 and AGS cells treated with or without 10 μM BAY11-7082 (NF-κB inhibitor) for 24 h. (**F**) Western blot and quantitation showed that ADAMTS16 overexpression promoted the phosphorylation of P65 in the nucleus. (**G**,**H**) Co-immunoprecipitation assays revealed that ADAMTS16 interacted with IκBα in HGC27 and AGS. (**I**) Immunofluorescence assays revealed that ADAMTS16 was co-localized with IκBα in HGC27 and AGS cell cytoplasm. (**J**) Schematic representation of IFI27 promoter organization and the corresponding luciferase reporter constructs pGL3-IFI27-WT, Mut1, and Mut2. Transcriptional start site, E1 exon1, and Luc luciferase. The blue and red bars indicate the binding sites of P65, including original and mutated sequences. (**K**) Dual-luciferase reporter assays were performed to analyze the activity of the pGL3-IFI27-WT, Mut1, and Mut2 constructs in HGC27 and AGS cells. Scale bar, 50 µm. The data are shown as means ± standard deviations. * *p* < 0.05, ** *p* < 0.01, *** *p* < 0.001, **** *p* < 0.0001. ns, not significant.

To identify the ADAMTS16-mediated signal transduction pathways that promote GC cell growth and invasion, we performed RNA-Seq and bioinformatics analyses of AGS-vector/ADAMTS16 cells. A total of 806 genes altered their mRNA expression, of which 363 were upregulated (ADAMTS16/vector) and 443 were downregulated (Figure S1). The top 20 most differently expressed genes are listed in Figure 3A. Among them, we focused on IFI27. Moreover, we also found a significant positive correlation between the mRNA expression of IFI27 and ADAMTS16 (Figure 3B). These findings suggest that IFI27 may play an important role in ADAMTS16-induced promotion of GC cell growth, migration, and invasion. Furthermore, GSEA analysis indicated that the HALLMARK_TNFA_SIGNALING_VIA_NFKB (Figure 3C) pathway was enriched, suggesting that ADAMTS16 promotes cell migration, invasion, and proliferation through the NF-κB/IFI27 axis.

To further examine how ADAMTS16 promotes GC cells carcinogenesis through the NF-κB/IFI27 axis, we performed a series of analyses. We investigated the changes in the levels of NF-κB pathway proteins (IκBα, p-IκBα, P65, and p-P65). Compared with control cells, ADAMTS16 overexpression led to an increase in the expression of p-IκBα, p-P65, and IFI27 and a decrease in the expression of IκBα in HGC27 and AGS, no significant change in the expression of P65 (Figure 3D). ADAMTS16 knockdown represented the opposite effects in MKN1 and SGC7901 (Figure 3D). Furthermore, the expression of IFI27 decreased (Figure 3E) when the HGC27 and AGS (vector/ADAMTS16) cell lines were treated with the NF-κB pathway inhibitor BAY11-7082. These results suggested that ADAMTS16 can activate the NF-κB pathway to upregulate IFI27.

In this study, ADAMTS16 overexpression upregulated nuclear phosphorylated P65, while ADAMTS16 knockdown significantly downregulated it (Figure 3F). Co-immunoprecipitation revealed that ADAMTS16 can bind to IκBα (Figure 3G,H). Immunofluorescence showed that ADMTS16 was co-localized with IκBα in HGC27 and AGS cytoplasm (Figure 3I). We also explored the possibility that P65 directly affects IFI27 gene transcription. We analyzed the transcription start site of IFI27 using the JASPAR database. The analysis identified two binding sites that P65 may occupy (Table S1). P65 binding appears to activate the expression of IFI27. To investigate this possibility using dual-luciferase reporter assays, we produced the luciferase reporter constructs (pGL3-IFI27-WT, Mut1, and Mut2; Figure 3J). The transcription levels of Mut1 and Mut2 were decreased in HGC27 and AGS cells compared with WT when co-transfected with pCDNA3.1-P65-3xFlag (HGC27: pGL3-IFI27-WT vs. pGL3-IFI27-Mut2, *p* < 0.05; AGS: pGL3-IFI27-WT vs. pGL3-IFI27-Mut2, *p* < 0.01; Figure 3K). This indicates that P65 could bind to the promoter region of IFI27 from −1618 to −1609 bp. These results suggest that ADAMTS16 induces the expression of IFI27 through the NF-κB pathway and possibly through direct transcriptional activation.

### 2.4. IFI27 Knockdown Reverses ADAMTS16-Induced Promotion of GC Cell Growth and Invasion

**Figure 4:**
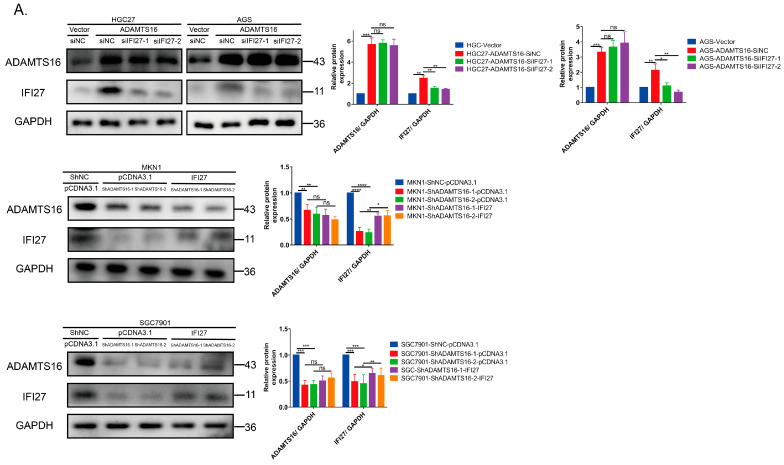

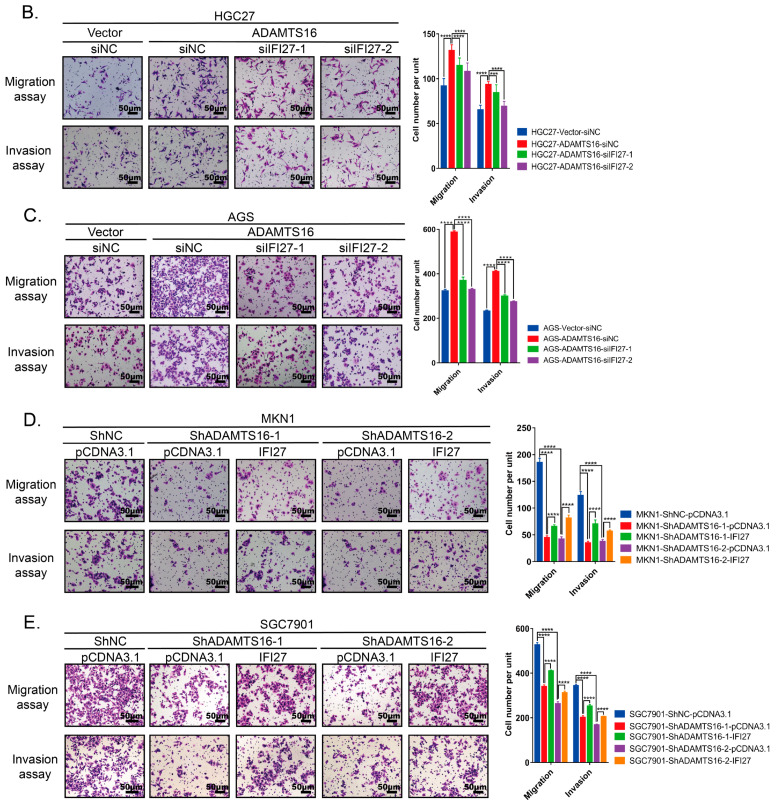

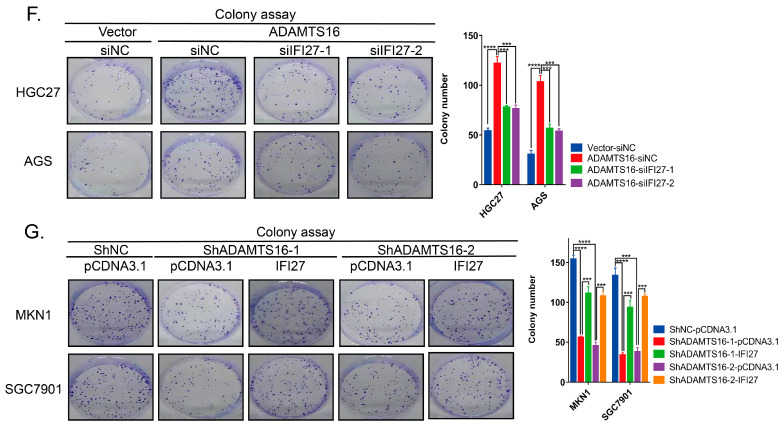
IFI27 protein knockdown reverses ADAMTS16-induced GC cell promotion. (**A**) Western blot and quantitation analysis of IFI27 in stably ADAMTS16-transfected HGC27 and AGS cells and stably knockdown ADAMTS16–transfected MKN1 and SGC7901 cells. (**B**–**E**) Transwell assays were performed to assess cell migration and invasion in the indicated cell lines (original magnification: 200×). (**F**,**G**) colony formation assays were performed to assess cell colony formation and proliferation ability in the indicated cell lines. Scale bar, 50 µm. The data are shown as means ± standard deviations. * *p* < 0.05, ** *p* < 0.01, *** *p* < 0.001, **** *p* < 0.0001. ns, not significant.

To further investigate the role of IFI27 in ADAMTS16-mediated promotion of GC cell growth and invasion, we knocked down IFI27 in HGC27 and AGS cells stably overexpressing ADAMTS16 and induced IFI27 overexpression in ADAMTS16 knockdown MKN1 and SGC7901 cells. The expression of IFI27 was validated by using western blotting (Figure 4A). As a consequence, migration and invasion assays were performed to further investigate the effect of IFI27 expression on GC cell. IFI27 knockdown inhibited the migration and invasion of HGC27 (migration: ADAMTS16-siNC vs. ADAMTS16-siIFI27-1, *p* < 0.0001; ADAMTS16-siNC vs. ADAMTS16-siIFI27-2, *p* < 0.0001; invasion: ADAMTS16-siNC vs. ADAMTS16-siIFI27-1, *p* < 0.001; ADAMTS16-siNC vs. ADAMTS16-siIFI27-2, *p* < 0.0001; Figure 4B) and AGS cells (migration and invasion: ADAMTS16-siNC vs. ADAMTS16-siIFI27-1, ADAMTS16-siNC vs. ADAMTS16-siIFI27-2, both *p* < 0.0001; Figure 4C). On the contrary, IFI27 overexpression promoted the migration and invasion of MKN1 and SGC7901 cells (ShADAMTS16-1-pCDNA3.1 vs. ShADAMTS16-1-IFI27, both *p* < 0.0001; ShADAMTS16-2-pCDNA3.1 vs. ShADAMTS16-2-IFI27, both *p* < 0.0001; Figure 4D,E). As expected, colony formation assay showed that knockdown of IFI27 significantly reversed colony formation ability of ADAMTS16 stable cell lines silencing IFI27 compared with control group cells (ADAMTS16-siNC vs. ADAMTS16-siIFI27-1; ADAMTS16-siNC vs. ADAMTS16-siIFI27-2; both *p* < 0.001, Figure 4F). Meanwhile, overexpression of IFI27 significantly rescued colony formation ability of silencing ADAMTS16 stable cell lines MKN1 and SGC7901 (ShADAMTS16-1-pCDNA3.1 vs. ShADAMTS16-1-IFI27; ShADAMTS16-2-pCDNA3.1 vs. ShADAMTS16-2-IFI27; both *p* < 0.001; Figure 4G).

### 2.5. High Expression ADAMTS16 Is Associated with Poorer Clinical Characteristics

**Figure 5:**
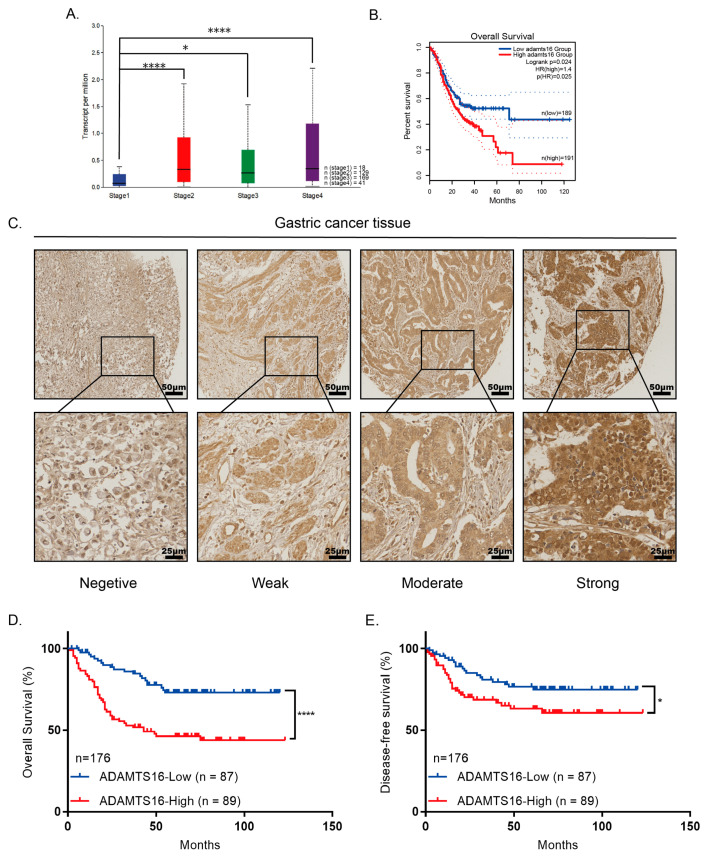
ADAMTS16 is upregulated in gastric cancer (GC) and is associated with a poor prognosis. (**A**) ADAMTS16 mRNA expression in advanced-stage GC tissue was higher than in early-stage GC tissue in TCGA. (**B**) GEPIA2 Kaplan–Meier plot of the effect of ADAMTS16 gene expression on GC patient survival. (**C**) Representative immunohistochemical staining image for ADAMTS16 protein expression level in GC. (**D**,**E**) Kaplan–Meier survival curves of GC patients on ADAMTS16 expression. Scale bar: 25 µm (**C** up), 50 µm (**C** down). The data are shown as means ± standard deviations. * *p* < 0.05, **** *p* < 0.0001.

By evaluating the expression of ADAMTS16 in a public dataset of GC patients, we observed that the mRNA levels of ADAMTS16 were significantly increased in advanced-stage GC tissue compared with early-stage GC tissue (Figure 5A). Meanwhile, the correlations between ADAMTS16 mRNA expression and the clinicopathological parameters of GC patients are summarized in Table S2. In GC, ADAMTS16 mRNA expression was significantly correlated with age (*p* = 0.004), invasion depth (*p* = 0.013), lymph node metastasis (*p* = 0.042), distance metastasis stage (*p* = 0.004), and TNM stage (*p* = 0.011). Kaplan–Meier analysis indicated that high ADAMTS16 expression was associated with poor prognosis (Figure 5B). To examine the impact of ADAMTS16 expression on GC progression, the associations between ADAMTS16 expression, survival data, and clinicopathological features of patients with GC were assessed. We next performed an IHC analysis of TMAs of 176 human GC tissues. A representative image of ADAMTS16 protein expression level is shown in (Figure 5C). All the samples that stained positively for ADAMTS16 exhibited a cytoplasmic localization, especially in those cases with high ADAMTS16 expression (Figure 5C). Those GC patients with high expression of ADAMTS16 presented not only a shorter OS (*p* < 0.0001) but also a shorter DFS (*p* < 0.05) compared with patients with low expression levels (Figure 5D,E). Here, GC patients with high ADAMTS16 expression showed a mean OS of 66 months (95% CI = 55–70 months), while patients with low ADAMTS16 expression presented a mean OS of 96 months (95% CI = 87–105 months). DFS of patients with high ADAMTS16 expression exhibited a mean of 82 months (95% CI = 71–94 months), while that of patients with low ADAMTS16 expression was significantly longer with a mean of 96 months (95% CI = 87–106 months).

In order to validate the prognosis potential of ADAMTS16 expression with respect to other clinicopathological characteristics, we performed a Cox proportional hazards model for both OS and DFS of GC patients. Univariate analyses for overall survival revealed high expression of ADAMTS16 as a risk factor (hazard ratio (HR) = 2.871; 95% CI: 1.698–4.853; *p* < 0.0001). Other clinicopathologic characteristics that associated significantly with shorter overall survival were TNM stage (HR = 4.080; 95% CI: 2.077–8.012; *p* < 0.0001), perineural invasion (HR = 2.573; 95% CI: 1.541–4.295; *p* = 0.0001), and vessel invasion (HR = 2.115; 95% CI: 1.305–3.427; *p* = 0.002) (Table S3). The clinical variables that associated significantly with reduced overall survival in the multivariate analysis were high expression of ADAMTS16 (HR = 2.285; 95% CI: 1.335–3.910; *p* = 0.003), TNM stage (HR = 2.487; 95% CI: 1.212–5.103; *p* = 0.013), and perineural invasion (HR = 1.884; 95% CI: 1.103–3.217; *p* = 0.020) (Table S3). The univariate analysis for DFS also revealed that patients with high expression of ADAMTS16 presented a higher risk of recurrence following surgery (HR = 1.867; 95% CI: 1.049–3.322; *p* = 0.034) (Table S4). Other pathological characteristics that were associated significantly with high risk of progression in the univariate analysis were TNM stage (HR = 4.897; 95% CI: 2.077–11.545; *p* < 0.0001), perineural invasion (HR = 3.075; 95% CI: 1.649–5.733; *p* < 0.0001), and vessel invasion (HR = 2.004; 95% CI: 1.141–3.522; *p* = 0.016) (Table S4). In the multivariate analysis, only TNM (HR = 3.200; 95% CI: 1.293–7.921; *p* = 0.012) stage and perineural invasion (HR = 2.182; 95% CI: 1.143–4.167; *p* = 0.018) remained statistically significant for higher risk of progression (Table S4). These results may indicate that ADAMTS16 is a potential biomarker to predict the prognosis of GC patients.

In view of these results, we verified that ADAMTS16 could be related to any of the pathological characteristics in our research (Table 1). High ADAMTS16 protein expression was significantly associated with invasion depth (*p* = 0.046), lymph node metastasis (*p* = 0.025), vascular invasion (*p* = 0.032), and pTNM stage (*p* = 0.006) (Table 1). These results suggest the aberrant ADAMTS16 expression as a deleterious effect in GC patient and support previous survival results.

## 3. Discussion

GC is one of the most common malignant gastrointestinal tumors in China [2]. Early diagnosis and advanced treatment strategies have made significant progress in the prognosis of GC patients [3,14,15,16], but the mortality rate of GC is still high [17,18]. The factors promoting GC development are intricate, and further research on the underlying molecular mechanisms is urgently needed. Recently, novel proteins called the ADAMTS proteins family have been discovered, and their expression has been observed in several types of tumors. ADAMTS protease family consists of 19 secreted zinc metalloproteases, whose substrates are primarily extracellular matrix (ECM) components [19]. ADAMTS proteins have been found to have both pro-tumor and anti-tumor effects in various cancer settings [20,21]. ADAMTS12 acts as a cancer promoter in colorectal cancer via activating the Wnt/β-catenin signaling pathway in vitro [22]. On the contrary, ADAMTS1 is an additional tumor suppressed protein, which was markedly decreased in lung, ovarian, and breast cancer [23,24,25].

As a member of the ADAMTS proteins family, ADAMTS16 was first revealed in the oncogene esophageal squamous cell carcinoma [12]. However, there is no research about ADAMTS16 on GC, until now, about whether it can reveal the latent molecular mechanisms. Firstly, we analyzed the data from TCGA, and the results revealed that the mRNA levels of ADAMTS16 were significantly increased in advanced-stage GC tissue compared to early-stage GC tissue. High ADAMTS16 expression was associated with a poor prognosis.

To further explore the concrete role of ADAMTS16 in GC progression, we employed a series of in vitro function assays. In our current study, aberrant ADAMTS16 promoted GC cells in vivo and in vitro by stimulating proliferation and restraining apoptosis. These findings were consistent with previously reported findings that high expression of ADAMTS16 promoted cancer cell proliferation and invasion ability in vitro [12].

Then we screened ADAMTS16 downstream effector and pathways by using RNA-Seq. Among the significantly different signaling pathways activated by ADAMTS16, we focused on the NF-κB pathway. It is universally known that activating sustained proliferation and metastasis are the typical hallmarks of cancers. We discovered that NF-κB related proteins including IκBα, p-IκBα (phosphor-IκBα), P65, and p-p65 (phosphor-P65) are influenced by ADAMTS16. Furthermore, ADAMTS16 was proven to interact with IκBα in cytoplasm by causing IκBα phosphorylation and degradation. Subsequently, the nuclear translocation of P65 was promoted. Meanwhile, we revealed that overexpression of ADAMTS16 promotes migration and invasion of GC cells in vitro, while knockdown decreases cell dispersion. Furthermore, we revealed that aberrant ADAMTS16 promoted GC cells in vivo and in vitro by stimulating proliferation and restraining apoptosis. Taken together, we not only identified a novel prognostic biomarker for GC, but also a potential common genetic pathway between GC and ADAMTS16.

**Figure 6:**
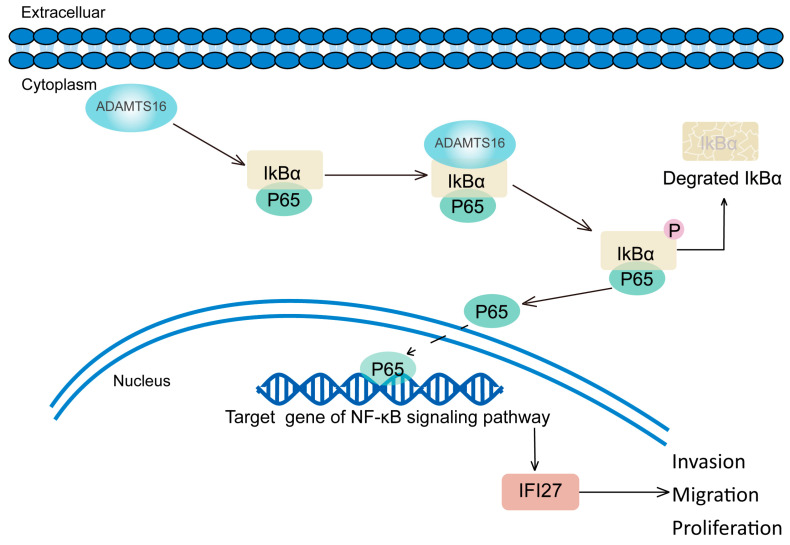
Model for the mechanism of ADAMTS16 in the development of GC.

We further investigated the target genes of ADAMTS16 in GC cell lines and its downstream molecular pathway. RNA-Seq and bioinformatics analyses of AGS-vector/ADAMTS16 cells showed that IFI27 was the most significant of the top 20 most differentially expressed genes. IFI27 (interferon alpha inducible protein 27), a member of the FAM14 family, is stably induced by interferon [26], and has been reported to regulate biological processes in numerous cancers [26,27,28]. In GC, Deng R et al. illustrated that IFI27 regulates tumor immunity via the canonical Wnt/β-catenin signaling pathway [29]. However, we revealed that IFI27 is regulated by ADAMTS16 in GC. Furthermore, the specific NF-κB pathway inhibitor BAY11-7082 suppressed IFI27 expression, indicating that the activation of the NF-κB/IFI27 signaling cascade is regulated by ADAMTS16. In previous studies, P65 is associated with the occurrence and development of various tumors [30,31,32,33]. The binding site between P65 and IFI27 was confirmed in our research. Subsequently, we found that IFI27 overexpression restored the invasion, migration, and proliferation abilities of GC cells to a certain extent. Therefore, we have reason to believe that ADAMTS16 promotes cell migration, invasion, and growth through the NF-κB/IFI27 axis (Figure 6). Accordingly, ADAMTS16 may function as a pro-tumor factor in GC development and progression in vivo, such that ADAMTS16 stimulated tumor growth in the xenotransplantation model in this study.

In addition, the prognostic role of ADAMTS16 in gastric cancer was also confirmed. In this study, clinicopathological analysis revealed that abnormal overexpression of ADAMTS16 was associated with a poor prognosis in human gastric tumors. The Cox proportional hazards model then revealed that high ADAMTS16 expression is an independent risk factor for poorer GC patient survival. Meanwhile, ADAMTS16 was significantly associated with the pathological characteristics including lymph node metastasis, local invasion, and vascular invasion. Our findings suggested that ADAMTS16 might be a potential biomarker for predicting the prognosis of GC patients, which could help us better understand the mechanism of GC development.

Numerous studies in recent years have revealed that the NF-κB pathway can regulate epithelial–mesenchymal transition (EMT) [34,35], influence the composition of the tumor microenvironment (TME) [36,37,38], and alter tumor resistance to chemotherapeutic drugs [39,40,41], thereby affecting tumor progression. For example, cancer-associated fibroblasts-derived IL-8 enhances chemoresistance via NF-κB activation in GC [42], gastric cancer cell-derived exosomes induce autophagy and pro-tumor activation of neutrophils via NF-κB by HMGB1/TLR4 interaction [43], and alpha B-crystallin promotes GC cells invasion and metastasis via NF-κB-induced EMT. Similar findings have been reported on the drug resistance and epithelial–mesenchymal transition of IFI27 in ovarian cancer [44,45]. In this study, a series of functional experiments have confirmed the regulation of ADAMTS16/NF-κB/IFI27 signaling axis in the development of gastric cancer, suggesting that ADAMTS16 can be regarded as a potential target for the treatment of gastric cancer. Whether ADAMTS16/NF-κB/IFI27 regulates EMT, TME, and tumor resistance still needs further study. Furthermore, members of the ADAMTS family are proteolytic enzymes of the extracellular matrix (ECM) and secretory proteins [44,45]. In recent years, the degradation of ECM has also been shown to promote tumor development [46,47], but the role of ADAMTS16 in gastric cancer remains unclear. Therefore, it is urgent for us to further study ADAMTS16 to promote the occurrence and development of GC, expecting to play a certain guiding role in the strategy of gastric cancer treatment.

## 4. Materials and Methods

### 4.1. Cell Lines

Five human GC cell lines (MKN1, BGC803, HGC27, SGC7901, and AGS) and the human normal gastric mucosal cell lines GES1 and HEK293T were obtained from the Type Culture Collection Cell Bank of the Chinese Academy of Sciences Committee (Shanghai, China). AGS and HEK293T were cultured in F12-K and DMEM, respectively, while the other cell lines were cultured in RPMI 1640 medium. All media were supplemented with 10% fetal bovine serum (FBS). The cells were incubated at 37 °C in a humidified atmosphere containing 5% CO_2_.

### 4.2. Plasmid Construction and Transfection

Expression plasmids for ADAMTS16 (NM_139056.4) and IFI27 (NM_001130080.3) were purchased from YouBio (Changsha, China) and WZ Bioscience (Jinan, China). The ADAMTS16 sequence was cloned into pCDH-GFP+Puro-3xFlag and pcDNA3.1(+)-Flag vectors. The IFI27 sequence was cloned into pLenti-CMV-MCS-SBP-3Flag-tRFP-F2A-Neo and pcDNA3.1(+)-HA vectors. The target sequences used for shRNA or siRNA gene-silencing plasmids were as follows: ShADAMTS16-1, CCGGGGAGGATAGCCGTAATGTTCTCGAGAACATTACGGCTATCCTCCTT.

TTTT; ShADAMTS16-2, CCGGGAGTATAAGTCTTGCTTACGGCATACTCGAGTATGCCGTAAGCAAGACTTATACTCTTTTTT; siIFI27-1, CTGCAGAGAAGAGAACCAT; siIFI27-2, TCTGGCTCTGCCGTAGTTT. The sequences were inserted into pLKO.1-TRC-copGFP-2A-PURO (WZ Biosciences, Jinan, China). The amplified vectors were transformed into competent *Escherichia coli* DH5α cells (TSINGKE, Beijing, China) and confirmed by sequencing. The vectors were isolated sequentially using an Endo-Free Plasmid Maxi Kit (Omega, Norcross, GA, USA). Transient transfection was performed using Lipofectamine 3000 reagent (Invitrogen, Carlsbad, CA, USA) according to the manufacturer’s instructions. For stable cell lines, 2 × 10^5^ GC cells were seeded on 6-well plates. When the cells had grown to 70–80% confluence, we replaced with fresh Opti-MEM (Invitrogen, Carlsbad, CA, USA) medium without FBS and used the ADAMTS16 or shADAMTS16 virus to infect the cells. After incubating for 72 h, the stable cells were obtained by puromycin screening. HGC27 and AGS were used for ADAMTS16 overexpression and IFI27 knockdown, whereas MKN-1 and SGC7901 were used for ADAMTS16 knockdown and IFI27 overexpression.

### 4.3. RNA Extraction and Quantitative Real-Time Polymerase Chain Reaction

An RNA Quick Purification Kit (EZBioscience, Guangzhou, China) was used to extract total RNA from cell lines and tissues following the manufacturer’s protocol. Reverse transcription PCR and qRT-PCR were performed using High-Capacity cDNA Reverse Transcription Kit (Applied Biosystems, Carlsbad, CA, USA) and SYBR Green Master Mix Kit (Applied Biosystems, Carlsbad, CA, USA) according to the manufacturers’ instructions.

The qRT-PCR primer sequences were as follows: ADAMTS16, 5′-CCGGCCGGTACAAATTTTCG-3′ (forward), 5′-AACAGCAGCTCCACAATCAGT-3′ (reverse); GAPDH, 5′-GACAGTCAGCCGCATCTTCTT-3′ (forward), 5′-AATCCGTTGACTCCGACCTTC-3′ (reverse); IFI27, 5′-TGCTCTCACCTCATCAGCAGT-3′ (forward), 5′-CACAACTCCTCCAATCACAACT-3′ (reverse).

### 4.4. Western Blot Assay

Cells were lysed with T-PER Tissue Protein Extraction Reagent (Thermo Fisher Scientific, Waltham, MA, USA) containing protease and phosphatase inhibitors (ApexBio, Houston, TX, USA). Nuclear and cytoplasmic proteins were separated using a Nuclear and Cytoplasmic Protein Extraction Kit (Beyotime Biotechnology, Shanghai, China) according to the manufacturer’s protocol. The protein concentration was quantitatively analyzed using a BCA Protein Quantitative Detection Kit (Servicebio, Wuhan, China) according to the manufacturer’s instructions. Protein samples were separated on SDS-PAGE gel and transferred to PVDF membranes (Millipore, Billerica, MA, USA). The PVDF membranes were blocked with 5% skimmed milk at room temperature for 1 h and incubated overnight with the primary antibody at 4 °C. They were then incubated with secondary antibodies at room temperature for 1 h and PVDF membranes were detected using a Meilunbio Pico Chemiluminescent Substrate (Meilunbio, Dalian, China). The membranes were then observed using a ChemiDoc Touch Imaging System (Bio-Rad, Hercules, CA, USA) and immunoblotted with the following primary antibodies: ADAMTS16 (OACD01415; 1:400; Avivasybio, San Diego, CA, USA), IFI27 (SAB1408588; 1:1000; Sigma, Darmstadt, Germany), P65 (#8242; 1:1000; Cell Signaling Technology, Boston, MA, USA), phospho-P65 (#3033; 1:1000; Cell Signaling Technology, Boston, MA, USA), IκBα (#4814; 1:1000; Cell Signaling Technology, Boston, MA, USA), and phospho-IκBα (#2859; 1:1000; Cell Signaling Technology, Boston, MA, USA). GAPDH (60004-1-Ig; 1:10,000; Proteintech, Wuhan, China) served as the internal control for total and cytoplasmic proteins, and H3 (384572; 1:1000; Zen BioScience, Chengdu, China) served as the internal control for nuclear proteins.

### 4.5. Migration and Invasion Assays

A transwell chamber with 8 µm pore size inserts (#353097, Falcon, New York, NY, USA) covered or uncovered with Matrigel (#356234, Corning, New York, NY, USA) was used to evaluate GC cell migration and invasion. In brief, 4 × 10^4^ cells were resuspended in 100 µL of serum-free medium and plated in the upper chamber, and 700 µL of medium containing 10% FBS was added to the lower chamber. After adequate incubation at 37 °C, the cells in the lower chamber were fixed with 4% paraformaldehyde and stained with crystal violet. Images of the cells in the lower chamber were captured under a microscope (Olympus, Japan), and cell counts were performed using ImageJ v1.52d. (National Institutes of Health, Bethesda, MD, USA).

### 4.6. Wound Healing Assays

A total of 4 × 10^4^ of stably transfected cells were seeded into a 12-well plate with Culture-Inserts 4 Well (Ibidi, Gräfelfing, Germany). After culturing overnight, the well was removed, and the cells were incubated with a serum-free medium for another 24–48 h. Images of the wound healing process were captured at every 2 h using Incucyte ZOOM (Essen BioScience, Michigan, MI, USA). To evaluate the cells’ wound healing ability, the percentage of wound closure was calculated using ImageJ (National Institutes of Health, Bethesda, MD, USA).

### 4.7. Colony Formation Assay

A total of 5 × 10^2^ of stably transfected cells were placed in a 6-well plate for colony formation assay. After adequate incubation at 37 °C for 10 to 14 days, the cells in the plate were fixed with 4% paraformaldehyde and stained with crystal violet. Cells in the 6-well plate were filmed by scanister (Canon, Tokyo, Japan) and calculated by ImageJ v1.52d. (National Institutes of Health, Bethesda, MD, USA).

### 4.8. Cell Proliferation Assays

A total of 1 × 10^3^ of stably transfected cells were placed in a 96-well plate for proliferation assays. Incucyte ZOOM (Essen BioScience, Michigan, MI, USA) was used to capture images every 2 h during incubation for 96–120 h and also used to calculate the cell occupation area in the plate according to the manufacturer’s instructions.

### 4.9. Apoptosis and Cell Cycle Assays

Stable cells were cultured in 6-well plates at a density of 1 × 10^5^ cells per well. After 48 h of culturing, the cells were harvested with or without a supernatant and stained using an Annexin V-APC/7-AAD apoptosis kit (MultiSciences, Hangzhou, China) and a cell cycle staining kit (MultiSciences, Hangzhou, China) according to the manufacturers’ instructions. Data were obtained using flow cytometry (Beckman Coulter, Brea, CA, USA) and analyzed with FlowJo v10.0 (BD Biosciences, Ashland, OR, USA) or CytExpert v2.4 (Beckman Coulter, Brea, CA, USA).

### 4.10. Tumor Xenotransplantation Model

All experiments were performed in accordance with the relevant guidelines and regulations of the animal care unit at Sixth Affiliated Hospital of Sun Yat-sen University. All in vivo experiments were approved by the Ethics Committee of the Sixth Affiliated Hospital of Sun Yat-sen University. Vector/ADAMTS16 HGC27 (1 × 10^6^) cells were injected subcutaneously into the left sides of female BALB/c nude mice (n = 5; 6 weeks old). The tumor weight was measured on day 28 after injection. IHC and TUNEL analyses were performed on the collected subcutaneous tumors.

### 4.11. NF-κΒ Inhibitor Treatment Assays

A total of 2 × 10^5^ HGC27 or AGS cells in 6-well plates were treated with 10 mM BAY11-7082 (SF0011; Beyotime Biotechnology, Shanghai, China) for 24 h. Cell pellets were lysed with T-PER and detected by western blot as described above.

### 4.12. RNA Sequencing Array and Bioinformatics Analysis

AGS-VECTOR/ADAMTS16 cells were analyzed using whole-transcriptome deep sequencing (RNA-Seq) on a BGISEQ-500 platform at the Beijing Genomics Institute. The data were analyzed using the database for annotation, visualization, and integrated discovery (DAVID, https://david.ncifcrf.gov/, accessed on 30 December 2020) and gene set enrichment analysis (GSEA) v4.1.0.

### 4.13. Co-Immunoprecipitation Assays (Co-IP)

Stably ADAMTS16-overexpressing and control cells were lysed and extracted (abs955; Absin, Shanghai, China) according to the manufacturer’s instructions. Primary antibodies were used to pull down the proteins that interacted with each other at 4 °C overnight. Protein detection was performed using western blot as described above after the extraction had been completed.

### 4.14. Immunofluorescence Assays (IF)

A total of 4 × 10^4^ cells were seeded into a 15-mm confocal dish after transient transfection with pCDNA3.1-ADAMTS16-Flag using Lipofectamine 3000 reagent as described above. After culturing for 24–48 h, the cells were fixed in 4% paraformaldehyde, and the cell membranes were penetrated with 0.25% Triton ×100 for 15 min. The cells were then blocked with 1% BSA at room temperature for 30 min and incubated overnight with primary antibodies at 4 °C. The primary antibodies were Flag (F1804; 1:1000; Sigma, Darmstadt, Germany) and IκBα (#4814; 1:200; Cell Signaling Technology, Boston, MA, USA). The cells were then incubated with secondary antibodies at room temperature for 1 h, and the nuclei were counterstained with DAPI for 5 min. Finally, the cells were observed, and images were captured using a confocal microscope (Carl Zeiss, Oberkochen, Germany).

### 4.15. Dual-Luciferase Reporter Assays

Plasmid pGL3-IFI27-WT/Mut1/Mut2 containing 1500–2000 bp upstream of two binding sites in the promoter region of IFI27 identified in public databases was purchased from IGEBIO (Guangzhou, China). The pGL3-IFI27-WT/Mut1/Mut2, pRL-TK, and pCDNA3.1-P65-3xFlag plasmids were co-transfected into HGC27 and AGS cells using Lipofectamine 3000 according to the manufacturer’s instructions. Using pGL3-IFI27-WT as a control, luciferase activity was measured using a Dual-Luciferase Reporter Assay System (Promega, Madison, WI, USA) according to the manufacturer’s protocol after 48 h of culturing.

### 4.16. Public Online Cancer Database Analyses

The public database the cancer genome atlas (TCGA, https://www.cancer.gov/about-nci/organization/ccg/research/structural-genomics/tcga, accessed on 25 January 2021) was used to search for differential ADAMTS16 expression in gastric tumors. GEPIA2 (http://gepia2.cancer-pku.cn/#index, accessed on 25 January 2021) was used for prognosis analysis of ADAMTS16 expression. The JASPAR database (https://jaspar.genereg.net/, accessed on 17 May 2021) was used to predict the binding site.

### 4.17. Patients and Tissue Samples

A total of 176 primary GC tissue samples were obtained from the Sixth Affiliated Hospital of Sun Yat-sen University, Guangzhou, China, from December 2007 to March 2012. The samples were embedded in paraffin blocks to construct tissue microarrays (TMAs) for immunohistochemistry (IHC). The patients were followed up until death or until 31 December 2018. Patients who were lost to follow-up were excluded from the analysis. The interval between the date of surgery and the date of death or the last follow-up visit was defined as overall survival (OS). The interval between the date of surgery and the date of local recurrence and/or metastasis was defined as disease-free (DFS).

### 4.18. Immunohistochemistry

ADAMTS16 expression was determined using a Biotin-Streptavidin-HRP-Detection-System (ZSGB-Bio, Beijing, China) for IHC staining. Briefly, specimens were incubated overnight with primary rabbit antibodies against ADAMTS16 (OACD01415; 1:50; Avivasybio, San Diego, CA, USA) at 4 °C, and the primary antibody diluent was used as a negative control. Finally, specimens were developed with 3,3-diaminobenzidine (DAB) for 75 s and counterstained with hematoxylin. ADAMTS16 expression in sections was evaluated independently by two pathologists using a semiquantitative scoring system. The semiquantitative scoring system was defined as follows: 0 (negative staining), 1 (weak staining), 2 (moderate staining), and 3 (strong staining) indicated the intensity of IHC staining; 1 (1–25%), 2 (26–50%), 3 (51–75%), and 4 (76–100%) was the percentage of stained cells. We calculated IHC score by multiplying the intensity of staining with the percentage of positive cells. The median (8.25) was used as the cutoff score.

### 4.19. Statistical Analyses

All data analyses were performed using IBM SPSS Statistics 21.0 software (IBM, New York, NY, USA). Figures were created using GraphPad Prism 7.0 software (GraphPad, San Diego, CA, USA). The results were expressed as means ± standard deviations. Comparisons between two groups were performed using Student’s t-test, the chi-squared test, or the Wilcoxon signed-rank test. The correlations between ADAMTS16 expression and clinicopathological characteristics were evaluated using the chi-squared test or Fisher’s exact test. Kaplan–Meier analysis (log-rank test) was performed to determine the correlation between ADAMTS16 expression and overall survival (OS) and disease-free survival (DFS). Cox stepwise multivariate regression analysis of prognostic factors was performed. Values of *p* < 0.05 were considered statistically significant in all tests.

## 5. Conclusions

In summary, this study indicates that ADAMTS16 plays a role as a tumor promoter and its upregulation is associated with a poor prognosis in GC. ADAMTS16 promotes GC cell invasion, migration, and proliferation. In terms of mechanism, ADAMTS16 interacts directly with IκBα cytoplasm to promote IκBα phosphorylated degradation and then P65 has been released into the nucleus, resulting in IFI27 upregulation, thereby promoting GC cell migration, invasion, and proliferation. Based on the clinical and biological significance of the ADAMTS16/NF-κB/IFI27 axis, ADAMTS16 can be considered as a GC prognostic biomarker and a potential therapeutic target.

## Figures and Tables

**Table 1:** Correlation between the expression of ADAMTS16 and clinicopathological characteristics of GC patients (n = 176).

Clinicopathological Characteristics	Low ADAMTS16 (n = 87)	High ADAMTS16 (n = 89)	*p* Value
Age			0.131
<60 years	48 (55.2)	38 (42.7)	
≥60 years	39 (44.8)	51 (57.3)	
Gender			0.918
Male	59 (67.8)	61 (68.5)	
Female	28 (32.2)	28 (31.5)	
Histologic type			0.369
Tubular or papillary adenocarcinoma	69 (79.3)	76 (85.4)	
Signet-ring cell carcinoma	14 (16.1)	7 (7.9)	
Mucinous adenocarcinoma	3 (3.4)	5 (5.6)	
Others ^a^	1 (1.1)	1 (1.1)	
Differentiation			1.000
Well-Moderately	11 (12.6)	11 (12.4)	
Poor	76 (87.4)	78 (87.6)	
Invasion depth			0.046
T1/T2	25 (28.7)	14 (15.7)	
T3/T4	62 (71.3)	75 (84.3)	
Lymph node metastasis			0.025
N0	29 (33.3)	16 (18.0)	
N+	58 (66.7)	73 (82.0)	
Distant metastasis			0.370
M0	79 (90.8)	77 (86.5)	
M1	8 (9.2)	12 (13.5)	
TNM Stage			0.006
I/II	37 (42.5)	20 (22.5)	
III/IV	50 (57.5)	69 (77.5)	
Perineural Invasion			0.999
Absent	43 (49.4)	44 (49.4)	
Present	44 (50.6)	45 (50.6)	
Vessel Invasion			0.032
Absent	59 (67.8)	46 (51.7)	
Present	28 (32.2)	43 (48.3)	

Statistical analyses were performed by the Pearson χ^2^ test. ^a^ Others: hepatoid adenocarcinoma and squamous carcinoma.

## Data Availability

All data are contained within the article or Supplementary Material.

## Supplementary Materials

The following supporting information can be downloaded at: https://www.mdpi.com/article/10.3390/ijms231911022/s1.
