# Supplementary material for: The Novel Protein ADAMTS16 Promotes Gastric Carcinogenesis by Targeting IFI27 through the NF-κb Signaling Pathway
Source: Int J Mol Sci. 2022 Sep 20;23(19):11022. doi: 10.3390/ijms231911022 (PMC9570124; doi:10.3390/ijms231911022)
Supplement: Supplementary file 1 [file ijms-23-11022-s001.zip › table s1.pdf]

Table S1 The sequence of binding site between P65 and IFI27 and its mutation

Name	Sequence
Homo-IFI27-WT	CCCGGGTGTGTCAACATAAAAAAAAAAAAAGAGAGAGAGGGAGACAAATCTTTAA
	GTAAAATCGTTTTACTTGGGAATAATACAAAAGAAGTAGGATTGCAATCCCACAACA
	TAAATATGGACCAGGGTGGCCTTTTTGTTTTGGAGAACAAAGGAAAAAGCTGGGG
	ATTTTTAGAGAAAGAGGCTGTTATGCAAGTTGTTCTGAAGGAAAGTTCAATGCAAT
	TTGTTTTATGAAAGTTATGCAAGTTGTTTTGAAAGAAAGTTTAATGGCTGTGATCCC
	ACAGCCGAAAGCTCACACCTGTAATCCCAGCAATTTGGGAGGCTGAGGCAGGAGG
	ATCACCTGACATCAGGAGTTTGAGACCAGCCGGGCCAACATGGTGAAACCCCATCT
	CTACTAAAAATACAAAAAATTAGCCGGGCATGGTGGCAGTTGCCTTTAATCCCAGCT
	ACTTGGGAGGCTGAGGCAGGAGAATCACTTGAATCACTCCACCGGGAGGTGGAG
	GTTGCAGTGAGCCGAGATTGCGCCATTGCCCTCCAGCCTGGGCAACAAGAGCAAA
	ACTCCATAAAAAAAAAAAAGAAAGGAAA GGGAAGCTT
Homo-IFI27-mut1	CCCGGGTGTGTCAACATAAAAAAAAAAAAAGAGAGAGAGGGAGACAAATCTTTA
	AGTAAAATCGTTTTACTTGGGAATAATACAAAAGAAGTAGGATTGCAATCCCACAA
	CATAAATATGGACCAGGGTGGCCTTTTTGTTTTGGAGAACAAAGGAAAAAGCTGG
	GGATTTTTAGAGAAAGAGGCTGTTATGCAAGTTGTTCTGAACCTTTCAAGTATGCA
	ATTTGTTTTATGAAAGTTATGCAAGTTGTTTTGAAAGAAAGTTTAATGGCTGTGATC
	CCACAGCCGAAAGCTCACACCTGTAATCCCAGCAATTTGGGAGGCTGAGGCAGGA
	GGATCACCTGACATCAGGAGTTTGAGACCAGCCGGGCCAACATGGTGAAACCCCA
	TCTCTACTAAAAATACAAAAAATTAGCCGGGCATGGTGGCAGTTGCCTTTAATCCCA
	GCTACTTGGGAGGCTGAGGCAGGAGAATCACTTGAATCACTCCACCGGGAGGTG

	GAGGTTGCAGTGAGCCGAGATTGCGCCATTGCCCTCCAGCCTGGGCAACAAGAG
	CAAAACTCCATAAAAAAAAAAAAAGAAAGGAAAGGGAAGCTT
Homo-IFI27-mut2	CCCGGGTGTGTCAACATAAAAAAAAAAAAAGAGAGAGAGGGAGACAAATCTTTAA
	GTAAAATCGTTTTACTTGGGAATAATACAAAAGAAGTAGGATTGCAATCCCACAACA
	TAAATATGGACCAGGGTGGCCTTTTTGTTTTGGAGAACAAAGGAAAAAGCTGGGG
	ATTTTTAGAGAAAGAGGCTGTTATGCAAGTTGTTCTGAAGGAAAGTTCAATGCAAT
	TTGTTTTATGAAAGTTATGCAAGTTGTTTTGAAAGAAAGTTTAATGGCTGTGATCCC
	ACAGCCGAAAGCTCACACCTGTAATCCCAGCAATTTGGGAGGCTGAGGCAGGAGG
	ATCACCTGACATCAGGAGTTTGAGACCAGCCGGGCCAACATGGACTTTGGGGTTC
	TCTACTAAAAATACAAAAAATTAGCCGGGCATGGTGGCAGTTGCCTTTAATCCCAGC
	TACTTGGGAGGCTGAGGCAGGAGAATCACTTGAATCACTCCACCGGGAGGTGGAG
	GTTGCAGTGAGCCGAGATTGCGCCATTGCCCTCCAGCCTGGGCAACAAGAGCAAA
	ACTCCATAAAAAAAAAAAAAGAAAGGAAA GGGAAGCTT

The blue and red bars indicate the binding sites of P65, including original and mutated sequences. The yellow bar and green bar represent 5’ region and 3’ region respectively.
