# Supplementary material for: The Novel Protein ADAMTS16 Promotes Gastric Carcinogenesis by Targeting IFI27 through the NF-κb Signaling Pathway
Source: Int J Mol Sci. 2022 Sep 20;23(19):11022. doi: 10.3390/ijms231911022 (PMC9570124; doi:10.3390/ijms231911022)
Supplement: Supplementary file 1 [file ijms-23-11022-s001.zip › table s2.pdf]

Table S2 Statistical association between ADAMTS16 mRNA expression with clinicopathological characteristics of TCGA (n=443).

**Clinicopathological characteristics**	**Low ADAMTS16 (n=407)**	**High ADAMTS16 (n=36)**	**P- value**
**Gender**			0.251
Male	265 (65.1%)	20 (55.6%)	
Female	142 (34.9%)	16 (44.4%)	
**Age**			0.004
< 65 years	193 (47.4%)	8 (22.2%)	
> 65 years	214 (52.6%)	28 (77.8%)	
**Invasion depth**			0.013
T1	22 (5.4%)	1 (2.8%)	
T2	81 (19.9%)	12 (33.3%)	
T3	190 (46.7%)	8 (22.2%)	
T4	110 (27.0%)	9 (25%)	
NA	4 (1.0%)	6 (16.7%)	
**Lymph node metastasis**			0.042
N0	125 (30.7%)	7 (19.4%)	
N1	108 (26.5%)	11 (30.6%)	
N2	80 (19.7%)	5 (13.9%)	
N3	83 (20.4%)	5 (13.9%)	
NA	10 (2.5%)	8 (22.2%)	
**Distance metastasis**			0.010
M0	364 (89.4%)	27 (75.0%)	
M1	23 (5.7%)	7 (19.4%)	

Mx	20 (4.9%)	2 (5.6%)	
**TNM stage**			0.011
I and II	179 (44.0%)	10 (27.8%)	
III and IV	212 (52.1%)	15 (41.7%)	
NA	16 (3.9%)	11 (30.6%)	
**Histologic type**			0.207
Adenocarcinomas	371 (91.2%)	35 (97.2%)	
Cystic, Mucinous and Serous Neoplasms	36 (8.8%)	1 (2.8%)	
**Lauren's Classfication**			0.729
Intestinal	77 (18.9%)	9 (25.0%)	
Diffuse	64 (15.7%)	5 (13.9%)	
Mixted	266 (65.4%)	22 (61.1%)	
**Grade**			0.071
low	10 (2.5%)	2 (5.6%)	
middle	148 (36.4%)	11 (30.6%)	
high	240 (61.1%)	23 (63.8%)	

Statistical analyses were performed by the Pearson $\chi 2$ test
