# Supplementary material for: The Novel Protein ADAMTS16 Promotes Gastric Carcinogenesis by Targeting IFI27 through the NF-κb Signaling Pathway
Source: Int J Mol Sci. 2022 Sep 20;23(19):11022. doi: 10.3390/ijms231911022 (PMC9570124; doi:10.3390/ijms231911022)
Supplement: Supplementary file 1 [file ijms-23-11022-s001.zip › table s3.pdf]

Table S3 Univariate and multivariate cox regression analyses for overall survival of gastric cancer (n=176).

Variable	Univariate analysis		Multivariate analysis	
	HR (95%CI)	P value	HR (95%CI)	P value
Age (≥ 60 years vs. <60 years)	1.103 (0.683-1.784)	0.688		
Gender (male vs. female)	1.274 (0.769-2.111)	0.347		
Histology (tubular/papillary/adenocarcinoma vs. the others)	0.769 (0.381-1.553)	0.464		
Differentiation (well/moderate vs. poor)	1.151 (0.550-2.410)	0.709		
TNM Stage (III/IV vs. I/II)	4.080 (2.077-8.012)	<0.0001	2.487 (1.212-5.103)	0.013
Perineural Invasion (present vs. absent)	2.573 (1.541-4.295)	<0.0001	1.884 (1.103-3.217)	0.020
Vessel Invasion (present vs. absent)	2.115 (1.305-3.427)	0.002	1.370 (0.833-2.271)	0.215
ADAMTS16 expression (high vs. low)	2.871 (1.698-4.853)	<0.0001	2.285 (1.335-3.910)	0.003
