# Supplementary material for: The Novel Protein ADAMTS16 Promotes Gastric Carcinogenesis by Targeting IFI27 through the NF-κb Signaling Pathway
Source: Int J Mol Sci. 2022 Sep 20;23(19):11022. doi: 10.3390/ijms231911022 (PMC9570124; doi:10.3390/ijms231911022)
Supplement: Supplementary file 1 [file ijms-23-11022-s001.zip › table s4.pdf]

Table S4 Univariate and multivariate cox regression analyses for disease free survival of gastric cancer (n=176).

Variable	Univariate analysis		Multivariate analysis	
	HR (95%CI)	P value	HR (95%CI)	P value
Age (≥ 60 years vs. <60 years)	0.843 (0.481-1.478)	0.552		
Gender (male vs. female)	1.277 (0.709-2.300)	0.416		
Histology (tubular/papillary/adenocarcinoma vs. the others)	1.001 (0.469-2.136)	0.998		
Differentiation (well/moderate vs. poor)	1.391 (0.551-3.551)	0.484		
TNM Stage (III/IV vs. I/II)	4.897 (2.077-11.545)	<0.0001	3.200 (1.293-7.921)	0.012
Perineural Invasion (present vs. absent)	3.075 (1.649-5.733)	<0.0001	2.182 (1.143-4.167)	0.018
Vessel Invasion (present vs. absent)	2.004 (1.141-3.522)	0.016	1.317 (0.737-2.354)	0.352
ADAMTS16 expression (high vs. low)	1.867 (1.049-3.322)	0.034	1.440 (0.799-2.595)	0.225
