# Supplementary figures and images for: The Novel Protein ADAMTS16 Promotes Gastric Carcinogenesis by Targeting IFI27 through the NF-κb Signaling Pathway
Source: Int J Mol Sci. 2022 Sep 20;23(19):11022. doi: 10.3390/ijms231911022 (PMC9570124; doi:10.3390/ijms231911022)

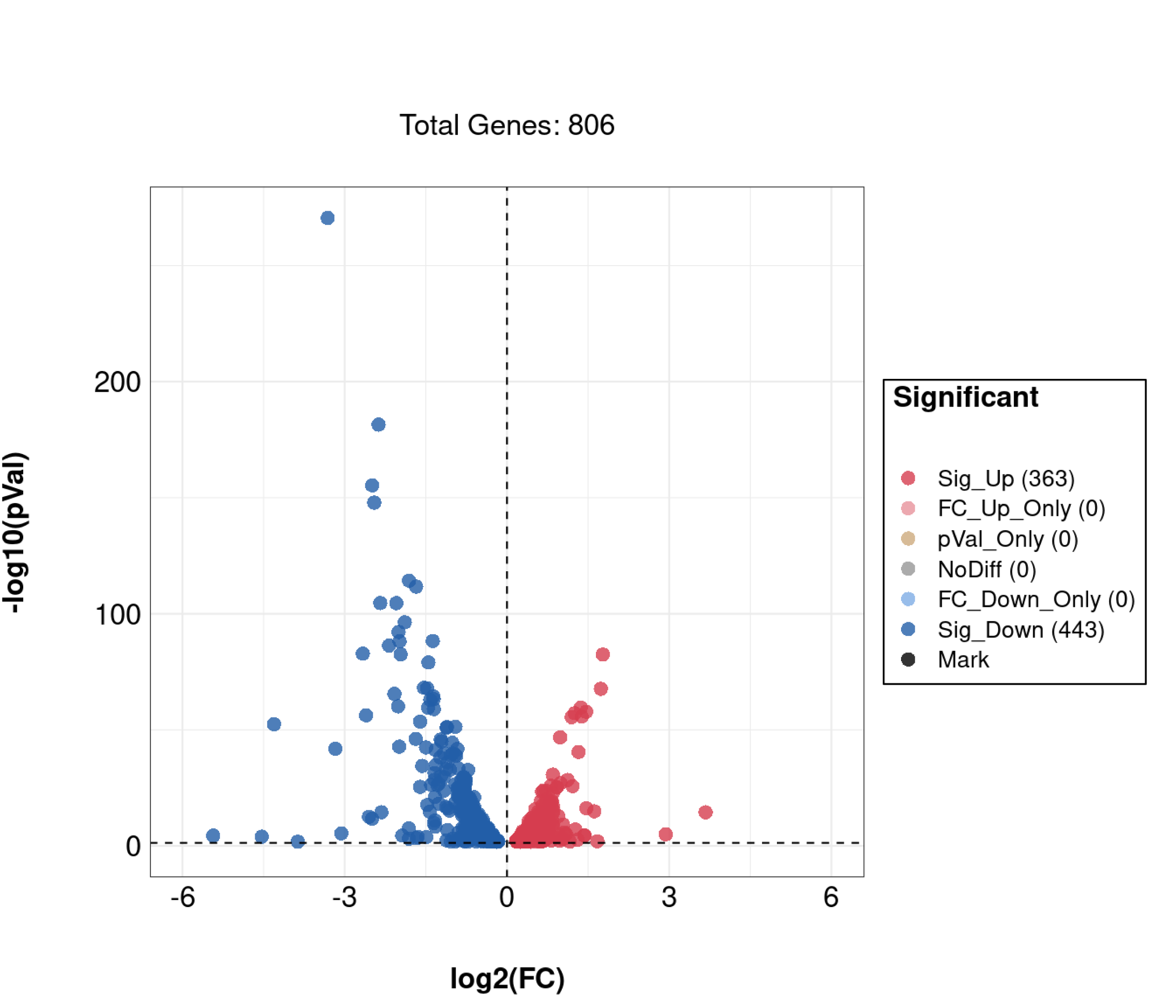

Supplement: Supplementary file 1 [file ijms-23-11022-s001.zip › figure s1.tif]
